# The free flap based on a single proximal perforator of the radial artery: ultrasonography study and clinical applications in reconstruction of soft tissue defects in finger

**DOI:** 10.1186/s40001-022-00702-2

**Published:** 2022-06-04

**Authors:** Guangzhi Wu, Zhan Zhang, Fan Zhang, Yiqun Zhang, Qianqian Wang, Wei Yu

**Affiliations:** Department of Hand Surgery, China-Japan Union Hospital of Jilin University, Xiantai Street 126, Changchun, 130033 People’s Republic of China

**Keywords:** Free flap, Radial artery flap, Digital soft tissue defects

## Abstract

**Objectives:**

To locate an anastomosable constant perforator of the radial artery on the proximal forearm using ultrasonography and describe the application of a free radial artery flap based on a single proximal perforator for the reconstruction of soft tissue defects in finger.

**Methods:**

In 20 forearms (ten right and ten left) from ten volunteers, the perforators in the proximal half of the forearm from the radial artery were visualized using ultrasonography. The free radial artery perforator flaps based on the single perforator were used to reconstruct digital soft tissue defects in four cases between October 2017 and May 2018.

**Results:**

Of the 20 forearms, an anastomosable perforator was consistently detected in the radial artery in the forearm’s proximal half. The perforator diameter was 0.7 ± 0.1 mm, and the pedicle length was 12 ± 3 mm according to ultrasonography. The perforator’s location was far from the elbow crease (8.8 ± 1.4 cm), and the relative distance of the perforator’s location from the elbow crease to the wrist crease was 37.2% ± 4.8%. In clinical cases, all flaps survived. Flap size ranged from 3.5 to 6.5 cm in length and 2.3–3.0 cm in width. Donor sites of the forearm were closed primarily in all cases. During a mean period of 12 months (8–14 months) follow-up, the average static 2-PD was 13.8 mm (10–18 mm) in the flap area, and the ROM of DIP was 35° (30–40°), PIP was 82° (45–110°), and MP was 85° (70–90°) of the affected finger. The mean Brief Michigan Hand Questionnaire (BMHQ) score was 72.9 (60.4–85.4) in the affected hand.

**Conclusions:**

An anastomosable perforator is consistently located on the radial artery in the proximal half of the forearm. The free radial artery flap based on this single perforator provides acceptable functional and cosmetic outcomes for reconstructing digital soft tissue defects. With the preservation of the forearm’s main vessel (radial artery), this flap provides another reliable option for hand surgeons to reconstruct small soft tissue defects in finger.

## Introduction

There are many options for reconstructing soft tissue defects in the fingers, including traditional or regional pedicled flaps and free flaps. In contrast, while the reconstruction of small digital defects (especially multiple digital defects) still presents a challenge to hand surgeons due to the apparent limitations of pedicled flaps (bulk, color, texture mismatch, and donor morbidity); free flaps have been recognized as the best reconstructive option for small digital soft tissue defects [1–3]. Various small free flaps can be harvested from the thenar, forearm (including venous flap), groin, partial toe, and plantar surface of the foot to reconstruct small soft tissue defects in finger [4–9]. Simultaneously, there is no ideal donor site for digital soft tissue defects reconstruction using the small free flap.

The standard radial forearm flap to reconstruct the soft tissue defects is familiar to plastic surgeons, but it is not suitable for digital defects due to the mismatch of vessel diameter. Only transferring a perforator of the radial artery for the flap to repair the digital soft tissue defects would be much more suitable than the standard one. Since the radial forearm flap was first described by Yang in 1978 [10], the anatomy of perforators of the radial artery in the forearm has been studied in cadaveric studies [11, 12]. This perforator cluster pedicle flap has been widely used in head or neck reconstruction as a free transfer [13], and in hand or elbow soft defect reconstructions as regional transfers [14, 15]. Nevertheless, either free or pedicle radial forearm flaps that are harvested using the traditional surgical technique are limited. The flap must sacrifice a major axial artery from the upper extremity. To preserve the radial artery, Lin and Omer first attempted to harvest the proximal perforator cluster flaps from the forearm’s radial artery to reconstruct head and hand defects in 2004 and 2005, respectively [16, 17]. Nevertheless, few studies are on the anastomosable perforators of the proximal radial artery in living patients. Duplex ultrasonography is a well-known method of evaluating the perforators for free flap transfer. This study aimed to locate a consistent anastomosable perforator of the radial artery on the proximal forearm using ultrasonography and then raise a free flap based on this single perforator in actual cases to assess the flap’s potential application for reconstruction of soft tissue defects in finger.

## Materials and methods

### Ultrasonography study

**Fig.1:**
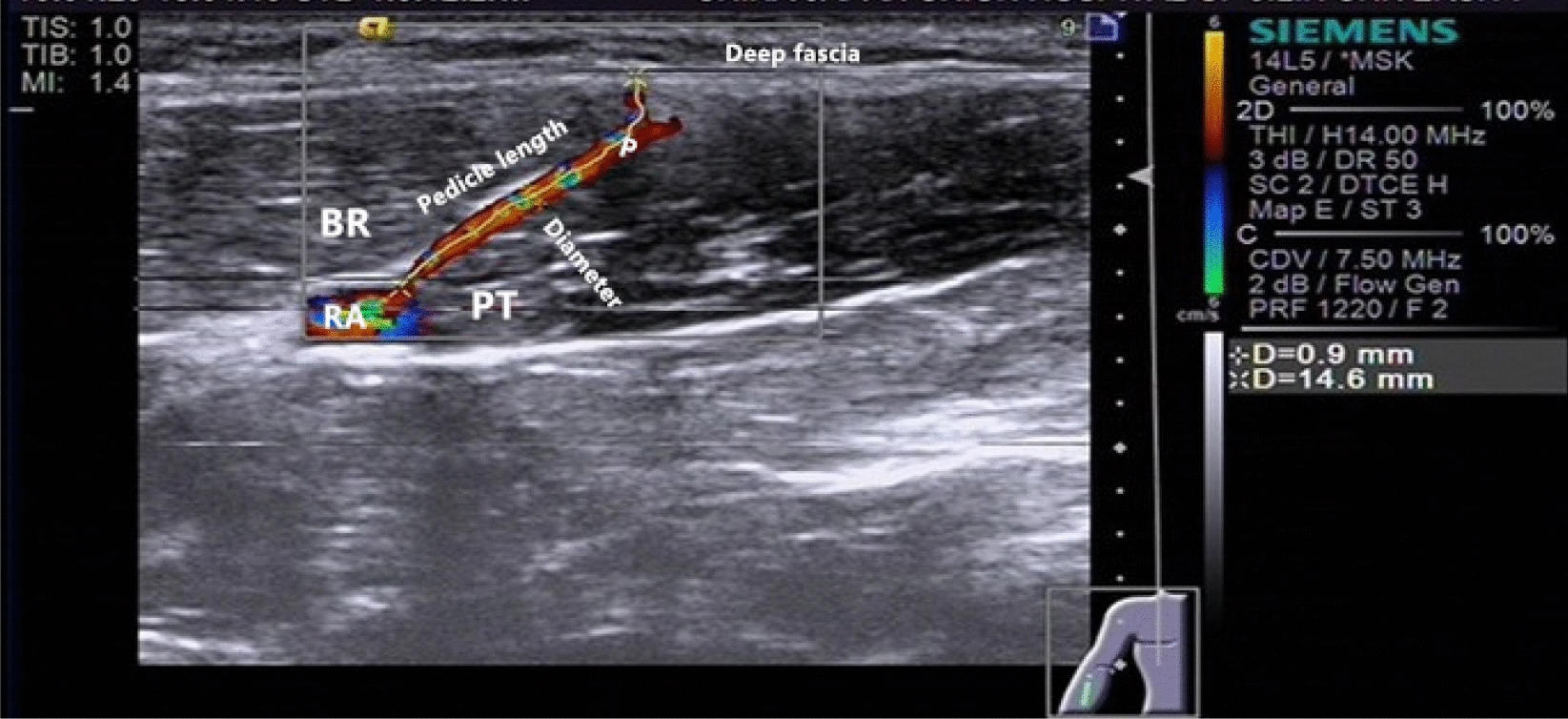
Perforator of Duplex ultrasound image: a perforator arising from the radial artery between the brachioradialis muscle and pronatorteres muscle in the proximal half of forearm. Diameter(+): the internal diameter of the perforator; Pedicle length(×): the pedicle distance from its origination of radial artery to deep fascia; *RA* radial artery; *P *perforator; *BR* brachioradialis muscle; *PT* pronatorteres muscle

**Fig.2:**
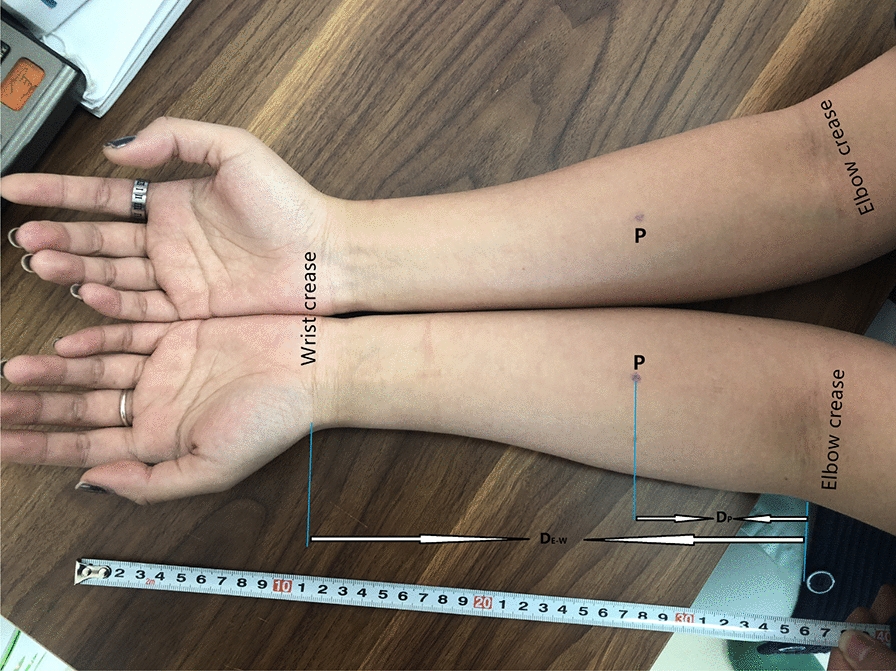
Location of the perforator of the radial artery in the proximal forearm. *P* perforator; *D*_*p*_ the distance from location of the perforator to the elbow crease; *D*_*E–W*_ the distance from the elbow crease to the wrist crease. Perforator Location = *D*_*p*_*/D*_*E–W*_* × 100%* the relative distance of the perforator location beyond the elbow crease

In 20 forearms (ten right and ten left) from ten healthy volunteers (five women and five men) with a mean age of 32 years (range 23–49 years), the perforators arising from the radial artery were identified in the proximal half of forearm using Doppler ultrasonography (Siemens, Acuson s2000; Siemens Medical Solutions USA, Inc; CA, USA). All ten volunteers were right-handed and had no histories of upper limb trauma, surgery, peripheral vascular disorders, or systemic metabolic diseases. When the radial artery and its perforator of the forearm were identified and located using a 14L5 high-frequency probe (5–14 MHz) in a supinated position, the internal diameter of the perforator and the pedicle length from radial artery to deep fascia were measured and recorded (Fig. 1). The distance from the perforator location to the elbow crease (*D*p) and the elbow crease's distance to the wrist crease (*D*_E–W_) was measured. The perforator location's relative distance on the ratio from the elbow crease to the wrist crease was calculated (*D*_p_/*D*_E–W_ × 100%) (Fig. 2). All data were recorded as mean ± standard deviation.

### Patients

Between Oct 2017 and May 2018, four patients (one man and three women) underwent reconstruction of digital soft tissue defects using the free radial artery perforator flaps based on the single perforator according to a protocol approved by the China–Japan Union Hospital. The ages of the patients ranged from 21 to 47 years. Soft tissue defects of digits were caused by various traumas (one friction injury, one stamping injury, and two crush injuries). All digital defects involved the tendon or exposed bone, including two digits with tendon defects; two digits were stumps of the fingers (one stump was a defect from the DIP, and one stump was a defect from the PIP). One patient had a single digital defect (left index finger), and the other three patients had two digital defects (two right middle and ring fingers; one right index and ring finger). The defects’ sizes ranged from 3.0 cm to 6.0 cm in length and 2.0 cm to 2.8 cm in width. The free radial artery perforator flap was used to reconstruct one digital soft-tissue defect (one right middle finger, one left index finger, two right ring finger, including two stumps of the fingers), other digital soft tissue defects were reconstructed by other free flaps or stump repair (two superficial palmar branch of radial artery free flaps, one stump repair).

### Surgical technique

Before the operation, the perforator’s location from the radial artery was determined and marked in the forearm's proximal half using ultrasonography. Under general anesthesia or brachial plexus block anesthesia, the patient was placed in a supine position, with upper extremity 90° abduction resting on a well-padded arm board. The operation was performed under pneumatic tourniquet control without limb exsanguination and under microscopic magnification to identify the perforator vessels better. After the devitalized tissue in the wound was debrided, the recipient's vessels were prepared in the defect. Depending on the size, the shape of the resulting defect, a free flap was drawn on the proximal forearm. The flap center was designed at the perforator’s location, entering into the deep fascia, which was marked using ultrasonography pre-operatively. The designed flap axis was the line from the middle point of the cubital fossa to the pulsation point of the radial artery at the styloid process. The flap elevation was started from the lateral border until the perforator vessel originating from the radial artery was determined between the brachioradialis and the pronator teres muscles. Then, the flap's medial border was incised, and retrograde dissection of the single perforator was conducted to the fascia, where the pedicle arose. Another subcutaneous vein was preserved and harvested into the designed flap to guarantee the flap's venous drainage. The branch of the lateral antebrachial cutaneous nerve was harvested into the flap if possible. However, there was no constant proper branch of the lateral antebrachial cutaneous nerve coursing into these small flaps, so nerve anastomosis was not routinely performed in this study.

The flap was then wholly elevated from the deep fascia. After releasing the tourniquet, the flap’s perfusion was confirmed, and the perforator pedicle was divided at its origin from the radial artery. One subcutaneous vein coursing across the flap was also harvested according to the length of the recipient’s vein. The raised flap was placed on the digital defect, and the perforator artery of the flap was anastomosed in an end-to-end fashion to the digital artery, the flap’s subcutaneous vein was anastomosed to the dorsal vein of the finger using 10–0 Prolene suture (Ethicon, USA) under the microscope. The flap margin was sutured to the defect margin, and the donor site was closed primarily.

### Postoperative management and follow-up

Standard postoperative free flap care and monitoring were performed for 7 days. Routine wound cleansing was accomplished using iodophor postoperatively. Low-molecular-weight heparin (5000 IU per day) and lower-molecular-weight dextran (500 mL per day) were used continuously to prevent thrombosis of the microsurgical anastomoses. All patients were instructed to avoid strenuous exercise of the operated fingers for 3 weeks, and one patient with the extensor tendon reconstruction was immobilized for 6 weeks. All skin sutures were removed 2 weeks after the operation. During this period, the survival of flap, early complications (e.g., infection, wound dehiscence, hematoma, and venous congestion) were intensively observed and recorded.

All patients were followed up regularly every 1–2 months. At the final follow-up, functional evaluation, including sensory recovery and range of motion (ROM) of the affected finger, were assessed and recorded. Sensory recovery was evaluated with static two-point discrimination (2-PD), and ROM of the distal interphalangeal joint (DIP), proximal interphalangeal joint (PIP) metacarpophalangeal joint (MP) were evaluated using a goniometer. The Brief Michigan Hand Questionnaire (BMHQ) was used for self-evaluating hand function. The BMHQ contained 12 questions with an original score of 1 (poor) to 5 (ideal) regarding six domains of hand function (overall function, daily life activities, work performance, pain, aesthetics, and satisfaction). The original scores were then calculated and recorded on a scale from 0 (poorest) to 100 (ideal function) [18].

## Results

### Ultrasonography

The anastomosable perforator was consistently detected from the radial artery in the proximal half of 20 forearms from ten healthy volunteers by ultrasonography. The internal diameter of the perforator was 0.7 ± 0.1 mm; the pedicle length from the radial artery to the deep fascia was 12 ± 3 mm; the distance from the perforator location to the elbow crease (*D*p) was 8.8 ± 1.4 cm, and the relative distance of the perforator location beyond the elbow crease (*D*_p_/*D*_E–W_ × 100%) was 37.2% ± 4.8% (Elbow crease was 0%; wrist crease was 100%) (Table 1).

**Table 1:** Location and ultrasonography findings of the radial artery perforator in the proximal forearm.(*n* = 20)

No.	Sex	Age	Side	Diameter (mm)	Pedicle length (mm)	*D*_p_/*D*_E–W_ (cm)	Perforator location (%)
1	Male	36	L	0.6	15	7.7/23.4	32.9
			R	0.7	15	9.0/23.5	38.3
2	Male	28	L	0.5	15	9.3/26.4	35.2
			R	0.5	20	10.8/25.1	43.0
3	Female	33	L	0.5	14	9.8/23.4	41.9
			R	0.5	13	6.7/23.5	28.5
4	Male	29	L	0.5	11	9.6/24.6	39.0
			R	0.8	14	8.5/24.4	34.8
5	Female	23	L	0.8	7	7.7/22.7	33.9
			R	0.6	14	8.6/22.8	37.7
6	Female	49	L	0.6	13	9.9/22.7	43.6
			R	0.8	10	8.3/22.7	36.6
7	Male	26	L	0.8	14	10.0/25.0	40.0
			R	0.9	15	9.3/23.9	38.9
8	Female	42	L	0.8	9	9.6/24.3	39.5%
			R	0.8	9	7.7/23.6	32.6
9	Male	24	L	0.7	11	11.1/25.0	44.4
			R	0.8	11	9.8/24.5	40.0
10	Female	34	L	0.7	5	5.6/21.1	26.5
			R	0.5	10	7.4/20.8	35.6
AVE (*x* ± *s*)		32 ± 8	–	0.7 ± 0.1	12 ± 3	8.8 ± 1.4/23.7 ± 1.3	37.2 ± 4.8

Diameter: the internal diameter of the perforator vessel of radial artery measured by e ultrasonography. Pedicle length: the pedicle distance from its origination of radial artery to deep fascia by ultrasonography. Perforator Location = *D*_p_/*D*_E–W_ × 100%: the relative distance of the perforator location beyond the elbow crease

*D*_p_: the distance from location of the perforator to the elbow crease; *D*_E–W_: the distance from the elbow crease to the wrist crease

### Clinical result

**Fig.3:**
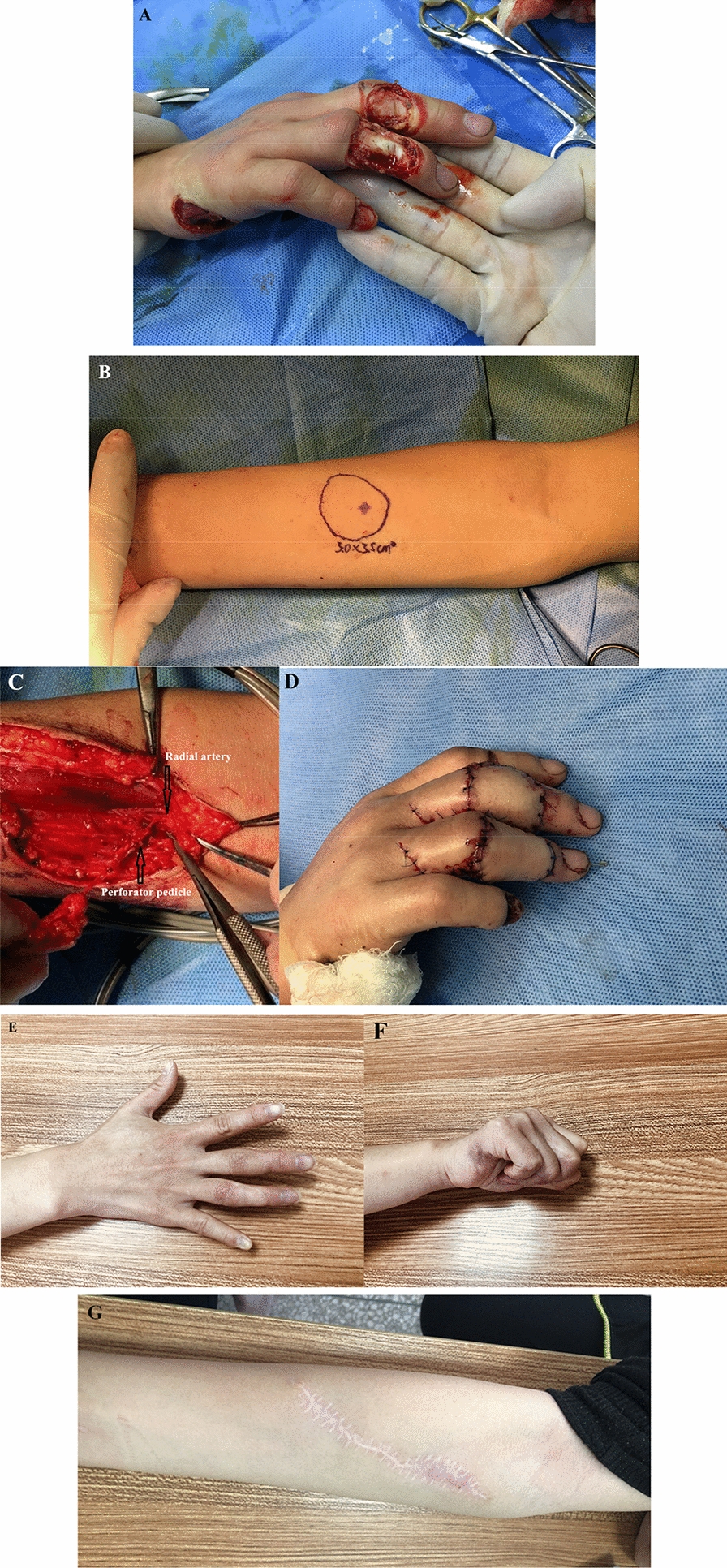
Case one-friction injury causing the multiple soft tissue defects in middle finger, ring finger and hypothenar area (**A**). A free radial artery perforator flap was designed (**B**) to reconstruct the defect in middle finger, basing on the single perforator in the proximal half of forearm (**C**).The appearance of the flap post-operatively immediately (**D**) and at 14 months after surgery (**E**), the functional result (**F**) and donor site scar (**G**) were acceptable. (Another free superficial palmar branch of the radial artery flap was designed to reconstruct the defect in ring finger; the full-thickness skin graft harvesting from forearm.to reconstruct the defect in hypothenar area.)

**Fig.4:**
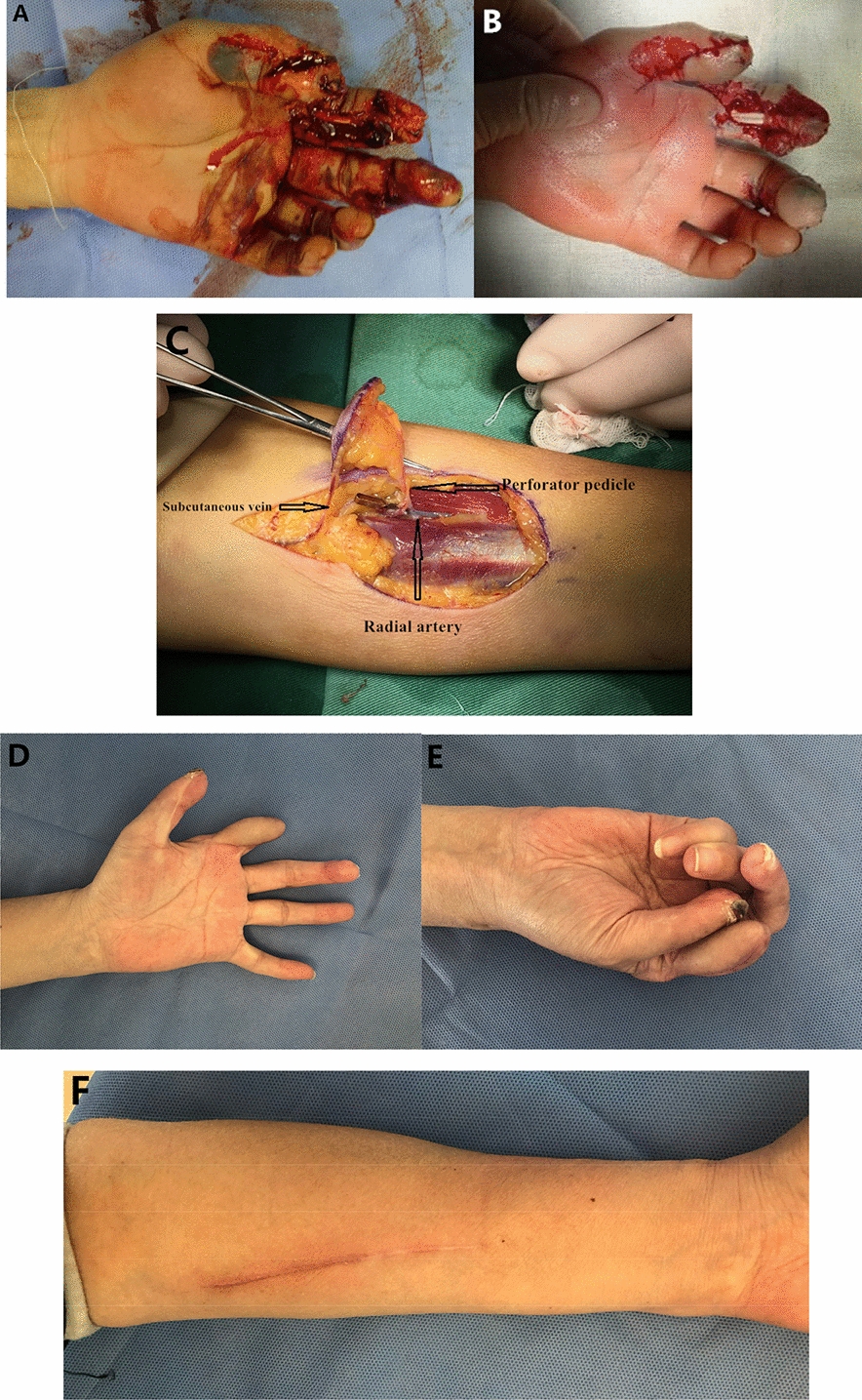
Case two-crush injury causing the volar soft tissue defects in index finger (**A**–**B**). A free radial artery perforator flap was designed, basing on the single perforator in the proximal half of forearm (**C**).The appearance of the flap at 12 months after surgery (**D**), the functional result (**E**) and donor site scar (**F**) were acceptable

**Fig.5:**
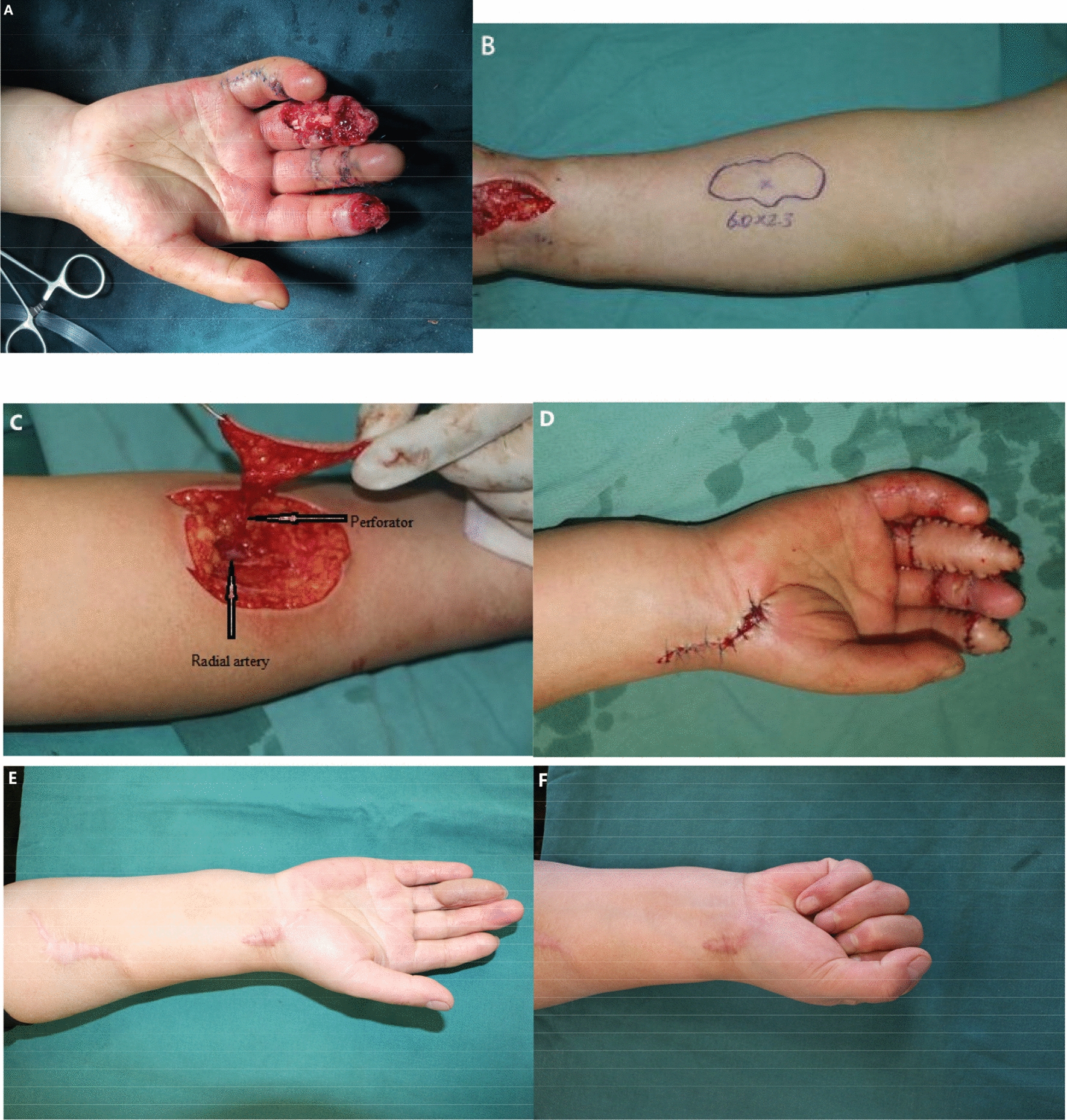
Case three-crush injury causing the volar soft tissue defects in index finger and ring finger (**A**). A free radial artery perforator flap was designed (**B**) to reconstruct the defect in ring finger, basing on the single perforator in the proximal half of forearm (**C**). The appearance of the flap post-operatively immediately (**D**) and at 8 months after surgery (**E**), the functional result (**F**) and donor site scar (**E**) were acceptable. (Another free superficial palmar branch of the radial artery flap was designed to reconstruct the defect in index finger.)

The cases of the flap are shown in Figs. 3, 4, and 5. All flaps were harvested from the ipsilateral forearm, and the donor sites were closed directly. The size of the flaps ranged from 3.5–6.5 to 2.3–3.0 cm. The diameter of the single perforator was 0.5–0.7 mm. There were no early complications (e.g., infection, wound dehiscence, hematoma, or venous congestion) in the cases, and all flaps were survived. During a mean period of 12 months (8–14 months) follow-up, no patient experienced cold intolerance, abnormal sensory, or scar pain. There were no functional impairments at the donor sites, and there were no secondary surgeries performed (tenolysis, scar adhesions, or flap debulking). The average static 2-PD was 13.8 mm (10–18 mm) in the flap area, and the average active ROM of the DIP was 35° (30–40°), PIP was 82° (45–110°), and MP was 85° (70–90°) on the affected finger. The data of patients and outcomes are shown in Table 2. The mean BMHQ total score for all patients was 72.9 (range 60.4–85.4); the overall hand function score of 78.1 (range 62.5–87.5); the activities of daily living score was 78.1 (range 62.5–100); the work performance score was 62.5 (range 50–75); the pain score was 62.5 (range 50–75); the aesthetics score was 68.8 (50–87.5), self-satisfaction score was 87.5 (range 75–100). The functional outcomes estimated by BMHQ of all patients are shown in Table 3.

**Table 2:** Data of patients and outcomes of the free radial artery perforator flap

No.	Sex	Age	Cause	Flap size (cm)	Perforator diameter (mm)	Recipient site	Follow-up time (months)	Complications	ROM (°)			S2-PD (mm)
									DIP	PIP	MP	
1	Female	21	Friction injury	3.5 × 3.0	0.6	Right middle finger	14	None	40	110	90	15
2	Female	47	Crush injury	5.0 × 3.0	0.6	Left index finger	12	None	–	–	70	12
3	Female	45	Crush injury	6.0 × 2.3	0.5	Right ring finger	8	None	30	90	90	10
4	Male	39	Stamping injury	6.5 × 3.0	0.7	Right ring finger	14	None	–	45	90	18

*ROM* range of motion; *DIP* distal interphalangeal joint; *PIP* proximal interphalangeal joint; *MP* metacarpophalangeal joint. *S2*-*PD* static 2 point discrimination

**–** represents the joint defect, so the data cannot be available

**Table 3:** Functional outcome of the free radial artery perforator flap estimated by BMHQ

No.	BMHQ Score						
	Overall function	ADL	Work performance	Pain	Aesthetics	Satisfaction	Total score
1	87.5	87.5	75	75	87.5	100	85.4
2	62.5	62.5	50	50	50	87.5	60.4
3	75	62.5	50	50	62.5	75	62.5
4	87.5	100	75	75	75	87.5	83.3
AVE (*x* ± *s*)	78.1	78.1	62.5	62.5	68.8	87.5	72.9

BMHQ (Brief Michigan Hand Outcomes Questionnaire) includes 12 questions divided into following 6 domains: overall function, daily life activities (ADL), work performance, pain, esthetics and satisfaction. The score of every item is normalized on a scale from 0 (poorest function) to 100 (ideal function) as the formula: 100 × (BMHQ original score −1)/4. The total score is the average of every item [18]

## Discussion

Trauma often leaves the hand surgeon with complex defects in the hand. Small defects (especially multiple digital defects) are challenging to reconstruct. The ideal reconstruction of digital soft tissue defects preserves the hand's function as much as possible while providing an aesthetic appearance both in the recipient and donor region. Among the various options for reconstructing small soft tissue defects in the finger, the free flaps have been recognized as the best reconstructive option [1–3].

Yang first described the radial forearm flap in 1978 [10]. This pedicle flap has been established in the plastic reconstructive field [13–15]. However, for either free or pedicle radial forearm flaps, the primary disadvantage is sacrificing a major artery in the upper extremity. In general, the radial artery’s sacrifice does not cause an ischemic problem unless the ulnar artery has been previously injured. Nevertheless, several studies reported severe complications (e.g., hypothenar hammer syndrome or dry gangrene of fingers) after harvesting a radial forearm flap [19, 20]. Based on the anatomical study of proximal perforators of the radial artery in the forearm [11, 12], Lin and Omer attempted to harvest this perforator free flap to preserve the radial artery [16, 17]. Few studies describe the proximal perforator’s use from the radial artery of the forearm for a free transfer.

Several radial artery perforators' analyses found that two main clusters of perforators (≥ 0.5 mm diameter; distal cluster, and proximal cluster) in the forearm could potentially be used for flap transfer [11, 14]. In a cadaveric anatomical study, Michel determined that the radial artery’s proximal cluster perforators were located 61.7% of the distance along with the radial styloid-to-lateral epicondyle interval. There are few studies on the anastomosable perforators of the proximal radial artery in living patients. In our study, an anastomosable perforator originating from the radial artery was detected in all proximal forearms. This perforator location was located at about 8.8 cm from the elbow crease. The relative distance was about 37.2%, along with the elbow crease-to-wrist crease interval, consistent with previous anatomical cadaver studies.

Furthermore, this perforator follows an intermuscular septal-type course between the brachioradialis and the pronator teres muscles. The pedicle length was 12 mm, and the internal diameter was 0.7 mm according to ultrasonography. These ultrasonography data showed that this perforator is consistently located at the radial artery in the forearm’s proximal half; designing a free flap based on this single perforator could be achieved. Furthermore, the perforator’s diameter can match the digit artery well in the reconstruction of the finger. The multiple superficial veins in the forearm can be harvested as an alternative donor vein for venous drainage. Our study also demonstrated the importance of ultrasonography preoperatively. Ultrasonography is a non-invasive and straightforward inspection method to locate a small perforator for designing a flap. Therefore, we suggest that perforator inspection and location using ultrasonography should be a routine examination before flap operation.

How much area could the free flap based on a single perforator of radial artery be done? According to the anatomical study of the radial artery’s proximal perforators, several perforators coming off the radial artery travel to the skin and form linking networks with one another along the radial artery as an axis (about 2-cm wide) [11]. This network of vessels between the fascia and the dermis ensures an adequate blood supply for designing a long flap (10–18 cm reported by Lin [16]). However, the width of this flap is limited (usually ≤ 4 cm). This shape features of an oblong flap is especially suited to reconstructing defects in long fingers.

There are some other advantages of this flap: first, single-perforator flap harvesting preserves the radial artery, avoiding the potential ischemic problem of the upper extremity. Second, the flap provides similar color and texture and aesthetic appearance to the finger. Finally, the operation can be performed in one stage in a single operative field.

The Brief Michigan Hand Questionnaire is a reliable and valid hand-specific instrument for evaluating hand function, including six distinct domains (overall function, daily life activities, work performance, pain, aesthetics, and satisfaction). The average original score in each domain is calculated to generate a score that is scaled from 0 (poorest) to 100 (ideal) [18]. In this study, the average total score of BMHQ in four patients was 72.9 (range 60.4–85.4), and the average satisfaction score was 87.5, and the overall function and ADL scores were 78.1. These data suggest that the hand function fully meets daily life needs, and the patients are satisfied with hand function after the surgery. The work performance score and the pain score were 62.5, possibly attributed to the double defects in two fingers or compound tissue damage (including tendon injury) that limit the affected hand's functional recovery.

In theory, the lateral antebrachial cutaneous nerve branch could be harvested into the flap and anastomosed to a cutaneous sensorial nerve of the recipient site. There are usually no proper nerve branches coursing into the small or medium-sized flap; furthermore, some studies found a high potential of spontaneous reinnervation after flap repair [21, 22]. Therefore, nerve anastomoses were not routinely performed in our cases, and we also obtained 13.8-mm mean static 2-PD in the flap area, which is acceptable sensory recovery. The average ROM of DIP, PIP, and MP were 35°, 82°, and 85°, respectively, in the affected finger, all of which are satisfactory for reconstructed fingers. The hand function and patient self-evaluations are consistent with previous studies using other free flaps (i.e., superficial palmar branches of the radial artery free flap) for reconstructing digital defects [23].

Some disadvantages of the radial artery proximal perforator free flap include non-concealed enough morbidity in the donor site, some bulkiness of the flap in the reconstructed finger, dissection, and anastomosis of microvessels. The aesthetics scores self-evaluated by the patients in our study were lower than 70.

There are several limitations in this study. First, we could not perform objective examinations for precise marking of the perforator directly and evaluate this single perforator's blood supply area. Second, the population sample was not large enough in the ultrasonography study and the clinical cases.

## Conclusions

There is an anastomosable perforator consistently located on the radial artery in the proximal half of the forearm. Preoperative detection and locating this perforator using ultrasonography can facilitate elevation of the flap. With consistent anatomy of the perforator and the satisfactory outcome in clinic application, the free radial artery flap based on this single perforator (preservation radial artery) is a reliable and useful option for reconstructing soft tissue defects in finger.

## Data Availability

The data sets used during the current study are available from the corresponding author upon reasonable request.

## References

[1] Liu Y, Jiao H, Ji X (2014). A comparative study of four types of free flaps from the ipsilateral extremity for finger reconstruction. PLoS ONE.

[2] Diaz-Abele J, Hayakawa T, Buchel E (2016). Anastomosis to the common and proper digital vessels in free flap soft tissue reconstruction of the hand. Microsurgery.

[3] Narushima M, Iida T, Kaji N (2016). Superficial circumflex iliac artery pure skin perforator-based superthin flap for hand and finger reconstruction. J Plast Reconstr Aesthet Surg.

[4] Noh SM, Kim JS, Lee DC (2008). Reconstruction of soft tissue defect of the finger with thenar free flap. Arch Plast Surg.

[5] Tancharoen C, Niumsawatt V, Ek EW (2014). Free distal volar forearm perforator flap: clinical application in digital reconstruction. ANZ J Surg.

[6] Wharton R, Creasy H, Bain C (2017). Venous flaps for coverage of traumatic soft tissue defects of the hand: a systematic review. J Hand Surge Eur Vol.

[7] Tare M, Ramakrishnan V (2009). Free ‘mini’ groin flap for digital resurfacing. J Hand Surg Eur.

[8] Orbay JL, Rosen JG, Khouri RK, Indriago I (2009). The glabrous palmar flap: the new free or reversed pedicled palmar fasciocutaneous flap for volar hand reconstruction. Tech Hand Up Extrem Surg.

[9] Koshima I, Urushibara K, Inagawa K, Hamasaki T, Moriguchi T (2001). Free medial plantar perforator flaps for the resurfacing of finger and foot defects. Plast Reconstr Surg.

[10] Yang G, Yuzhi G (1978). Forearm free skin flap transplantation. Natl Med J China.

[11] Saint-Cyr M, Mujadzic M, Wong C (2010). The radial artery pedicle perforator flap: vascular analysis and clinical implications. Plast Reconstr Surg.

[12] Onode E, Takamatsu K, Shintani K (2016). Anatomical origins of radial artery perforators evaluated using color doppler ultrasonography. J Reconstr Microsurg.

[13] Tornero AOJ, Cruztoro AOP, Farré A (2014). Free radial forearm flap in head and neck: our experience. Acta Otorrinolaringol Esp.

[14] Tiengo C, Macchi V, Porzionato A (2007). The proximal radial artery perforator flap (PRAP-flap): an anatomical study for its use in elbow reconstruction. Surg Radiol Anat.

[15] Koshima I, Narushima M, Mihara M (2010). The radial artery perforator-based adipofascial flap for coverage of the dorsal hand. Color Atlas Burn Reconstr Surg.

[16] Lin JY, Cheng MH, Wei FC (2006). Proximal Forearm Flap Based on a Septocutaneous Vessel from the Radial Artery. Plast Reconstr Surg.

[17] Ozkan O, Akyürek M, Coşkunfirat OK (2005). The free radial artery septal perforator vessel-based flap. Plast Reconstr Surg.

[18] Waljee JF, Kim HM, Burns PB (2011). Development of a brief, 12-item version of the michigan hand questionnaire. Plast Reconstr Surg.

[19] Jones BM, O’Brien CJ (1985). Acute ischemia of the hand resulting from elevation of a radial forearm flap. Br J Plast Surg.

[20] Heller F, Wei W, Wei FC (2004). Chronic arterial insufficiency of the hand with finger tip necrosis 1 year after harvesting a radial forearm free flap. Plast Reconstr Surg.

[21] Sinis N, Lamia A, Gudrun H (2012). Sensory reinnervation of free flaps in reconstruction of the breast and the upper and lower extremities. Neural Regen Res.

[22] Speidel EM (2000). Sensory recovery of innervated and non-innervated radial forearm free flaps: functional implications. J Reconstr Microsurg.

[23] Fan ZQ, Yu BF, Zeng Q (2019). The free neurovascular transverse wrist crease flap for repairing soft tissue defects of the fingers: clinical outcomes of multiple centers. J Orthop Surg Res.

